# Supplementary figures and images for: Effect of Humic Acid on Soil Physical and Chemical Properties, Microbial Community Structure, and Metabolites of Decline Diseased Bayberry
Source: Int J Mol Sci. 2022 Nov 25;23(23):14707. doi: 10.3390/ijms232314707 (PMC9738081; doi:10.3390/ijms232314707)

A

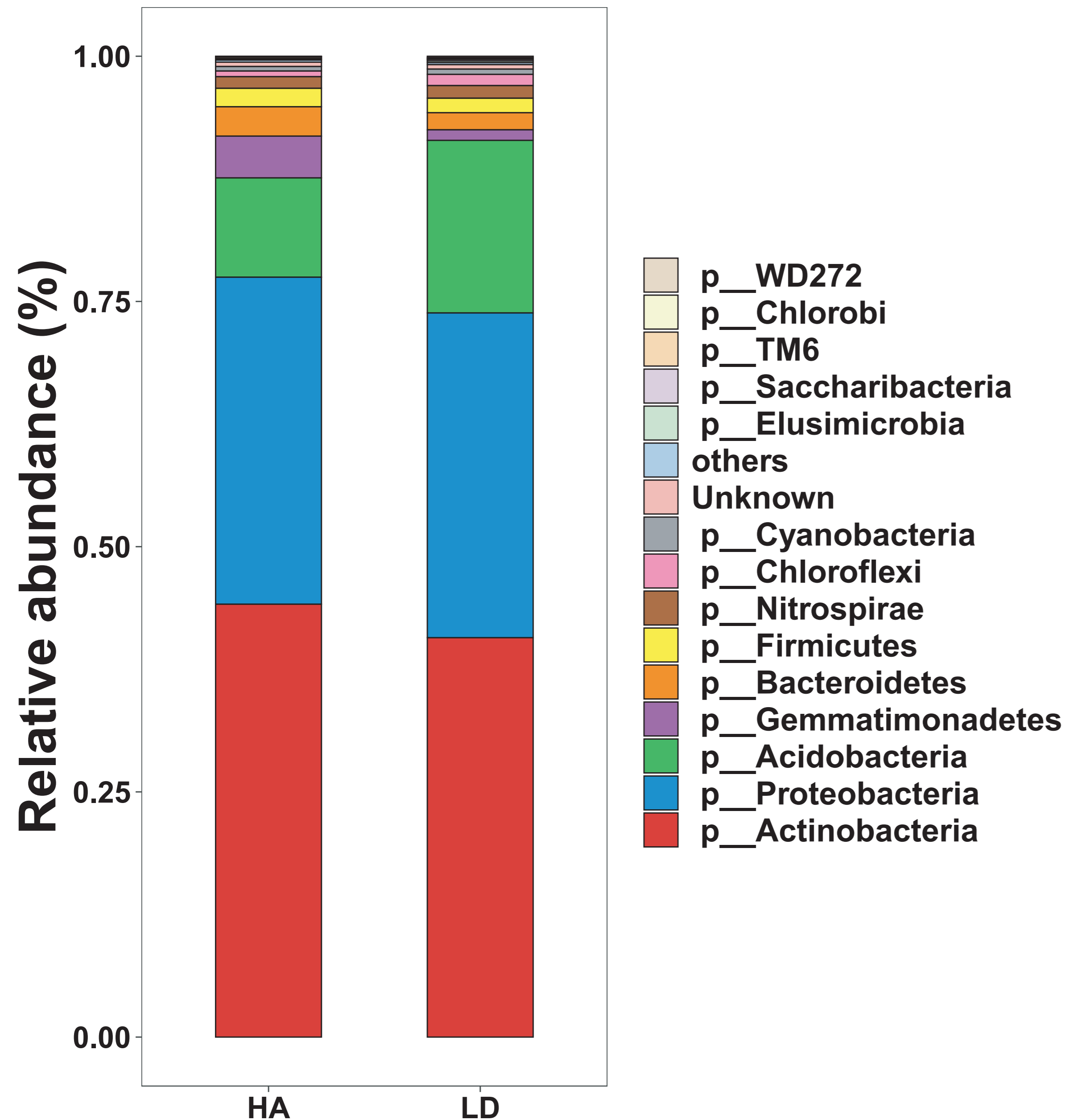

B

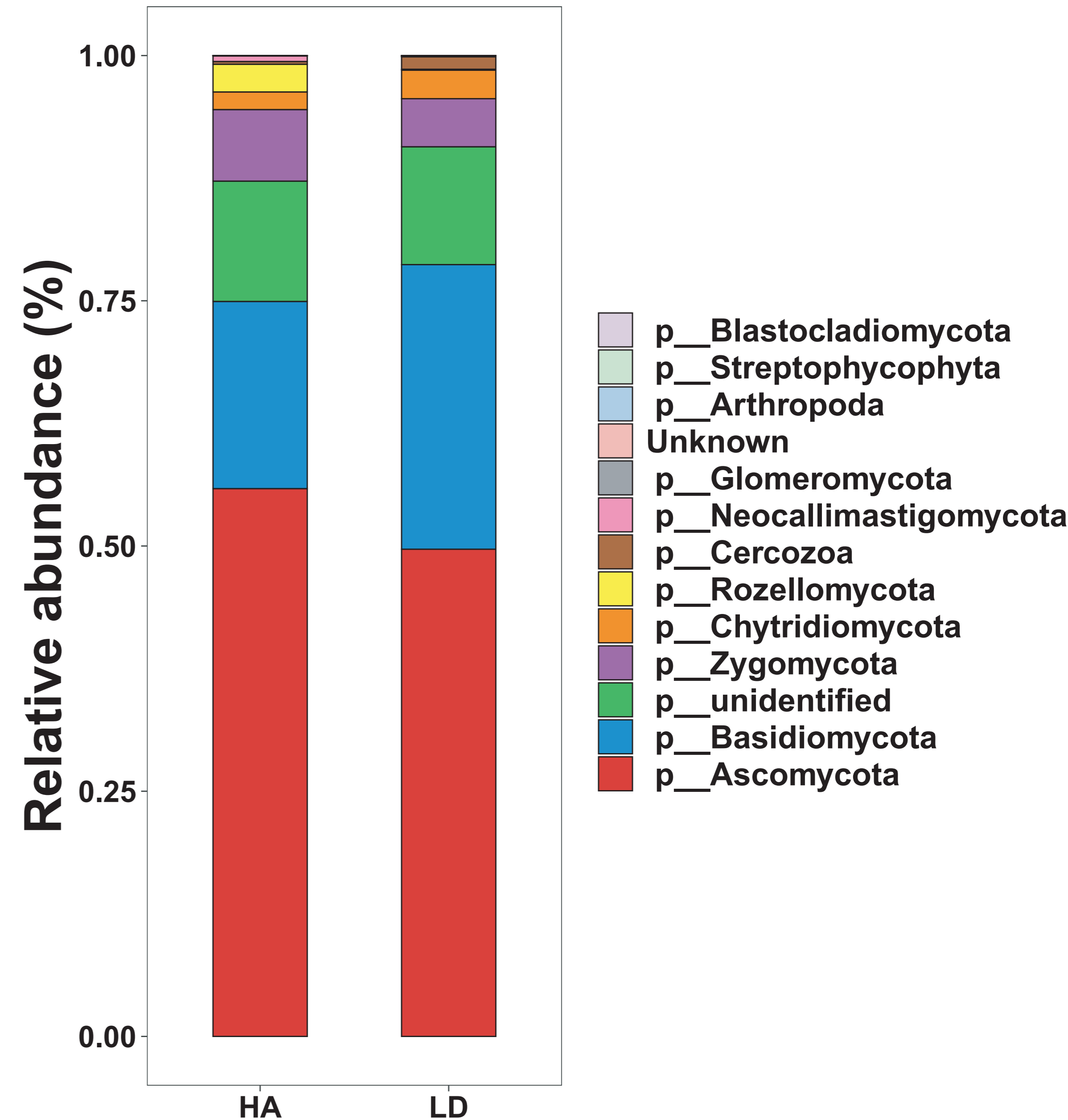

Supplement: Supplementary file 1 [file ijms-23-14707-s001.zip › ijms-2006527-supplementary/Supplementary files/Figure S1 phylum.pdf]

**A**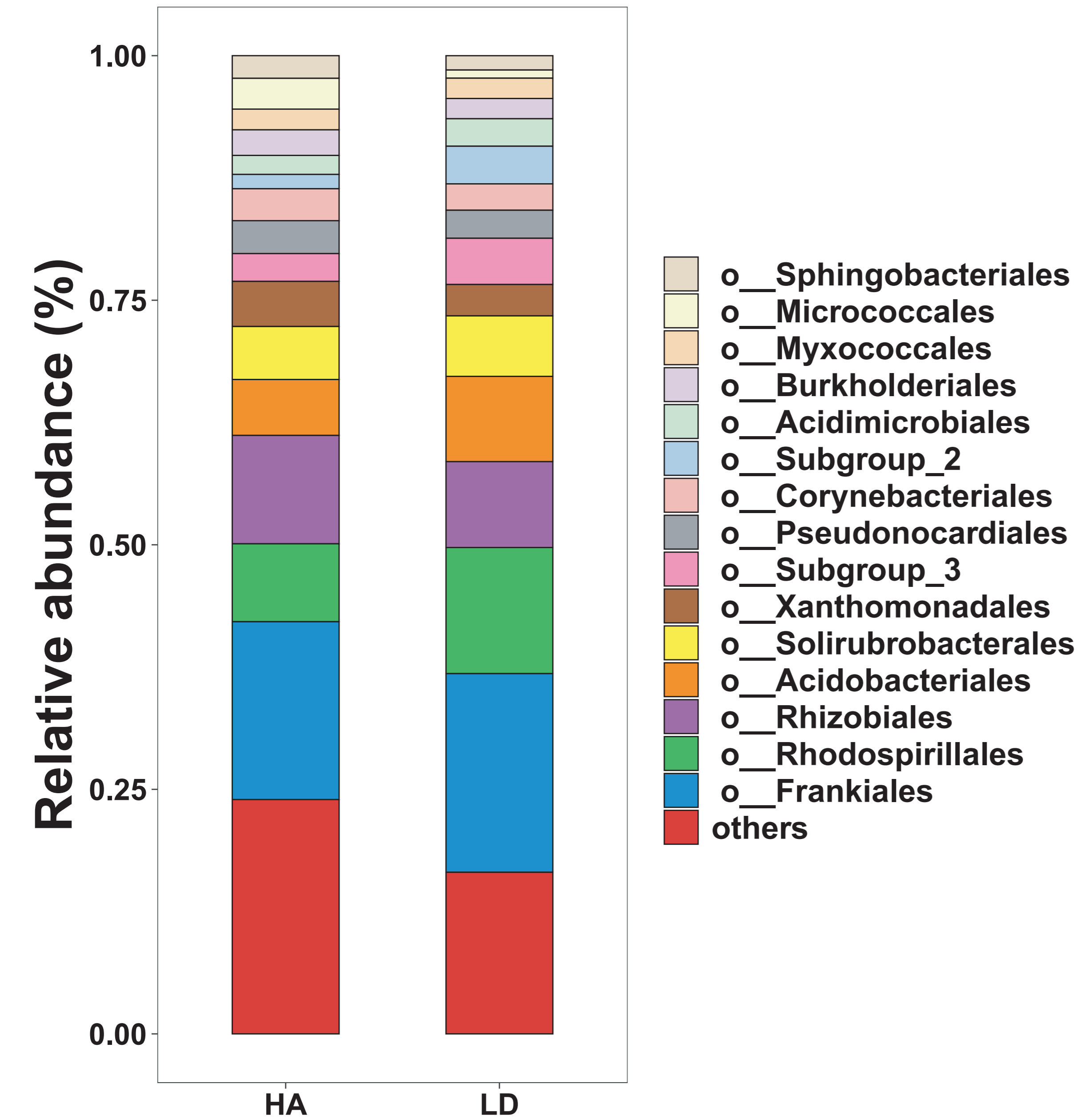**B**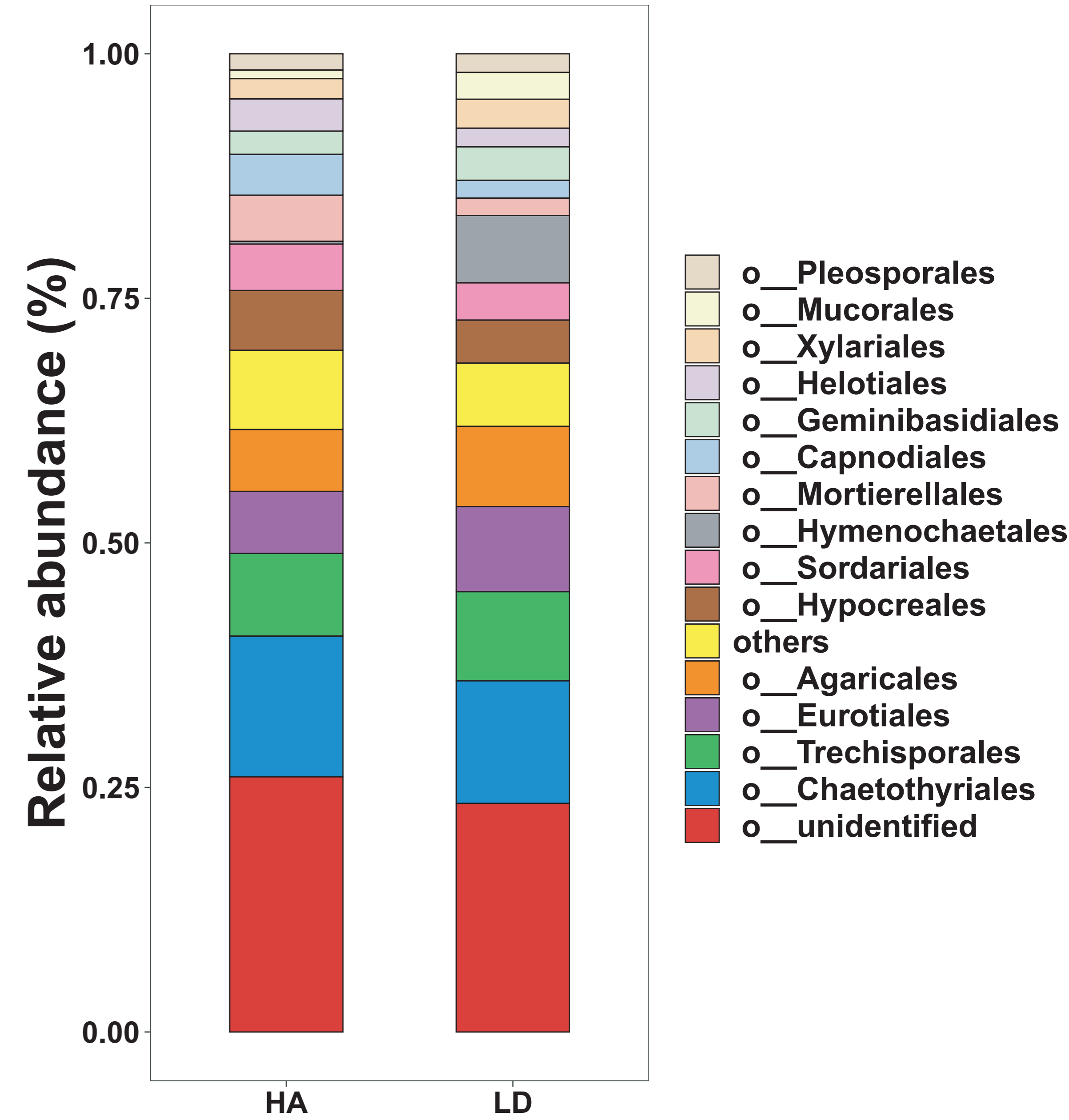

Supplement: Supplementary file 1 [file ijms-23-14707-s001.zip › ijms-2006527-supplementary/Supplementary files/Figure S2 order.pdf]

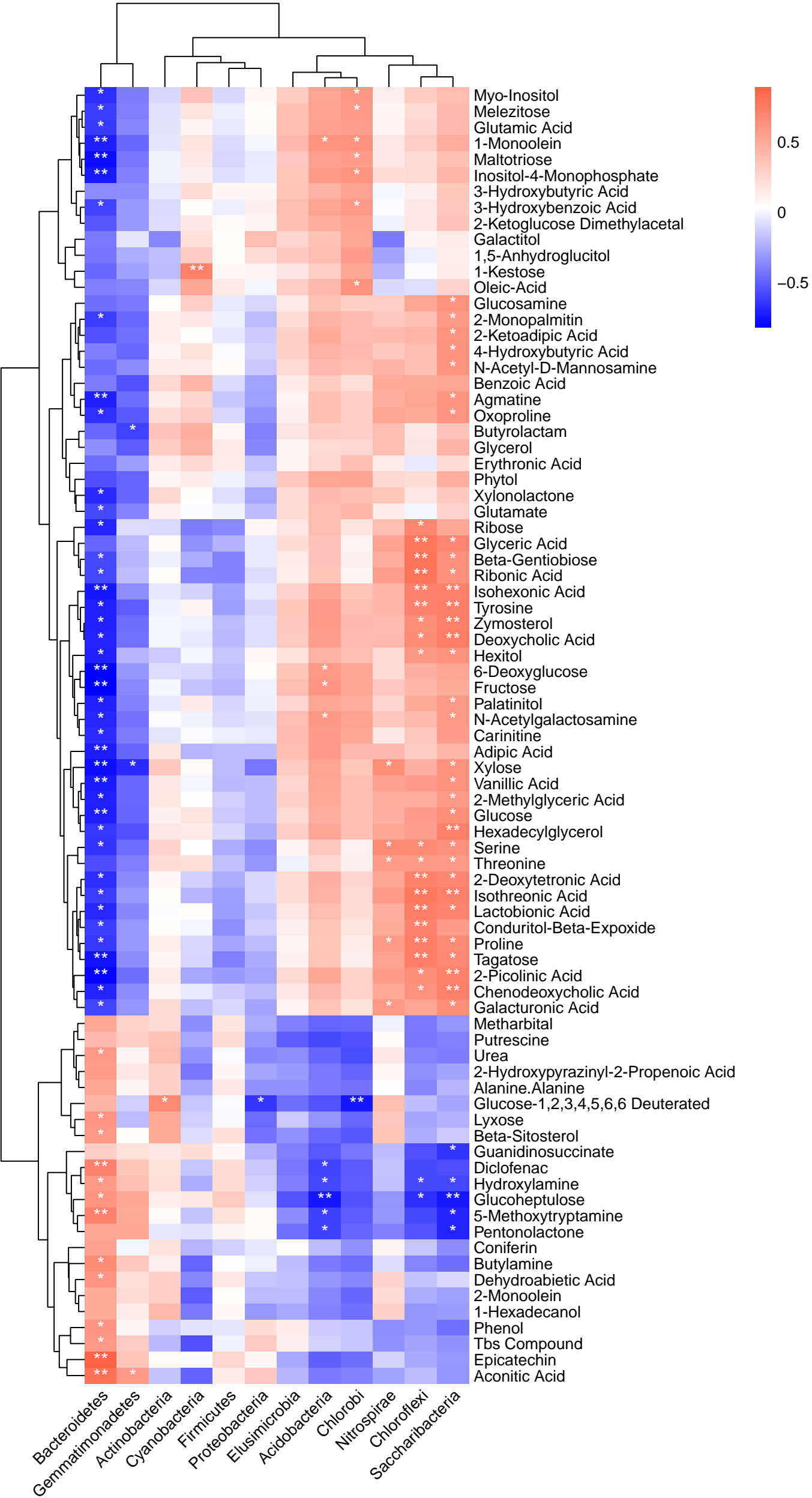

Supplement: Supplementary file 1 [file ijms-23-14707-s001.zip › ijms-2006527-supplementary/Supplementary files/Figure S3 16s_phylum_cor_heatmap.pdf]

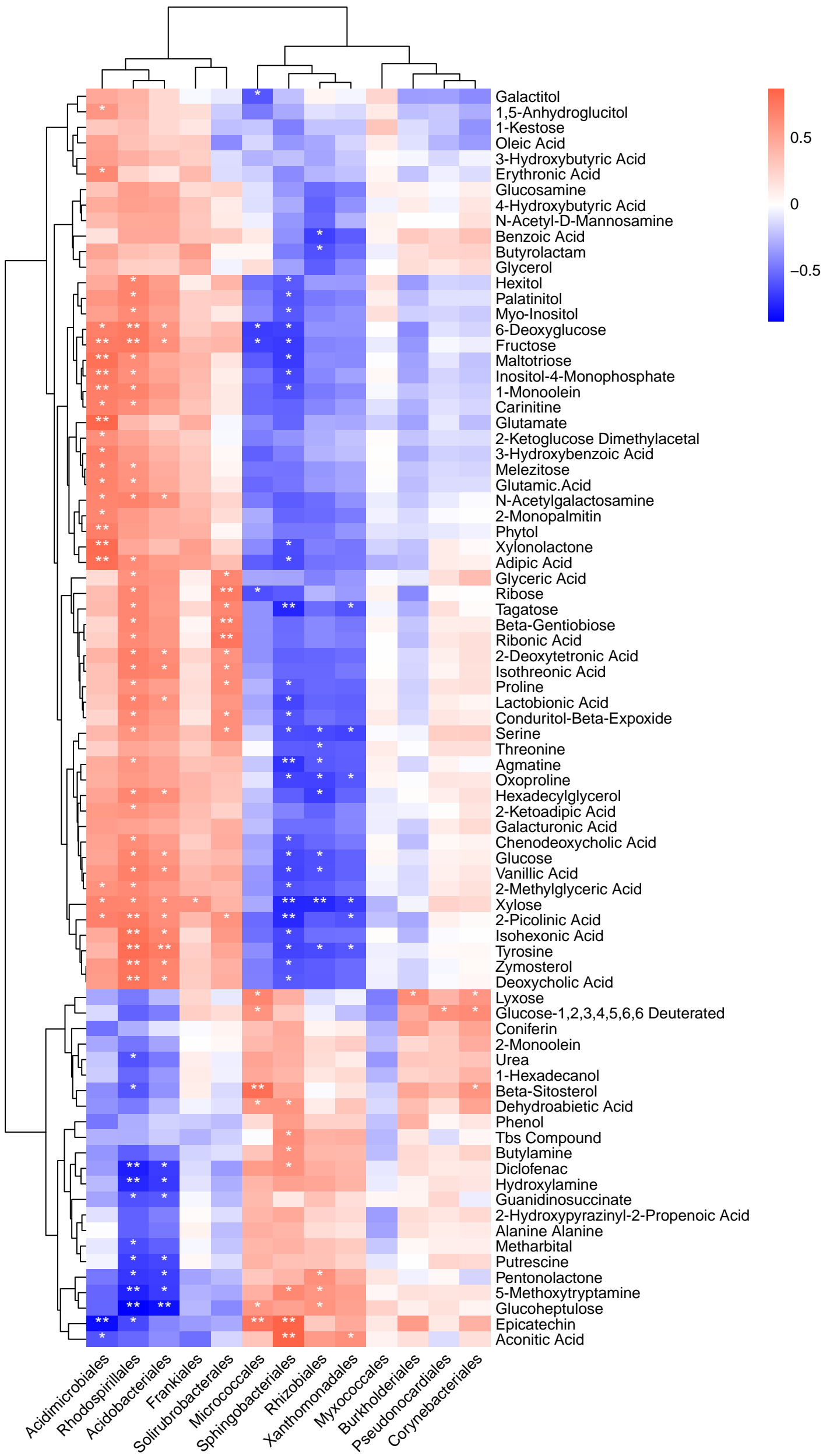

Supplement: Supplementary file 1 [file ijms-23-14707-s001.zip › ijms-2006527-supplementary/Supplementary files/Figure S4 16s_order_cor_heatmap.pdf]

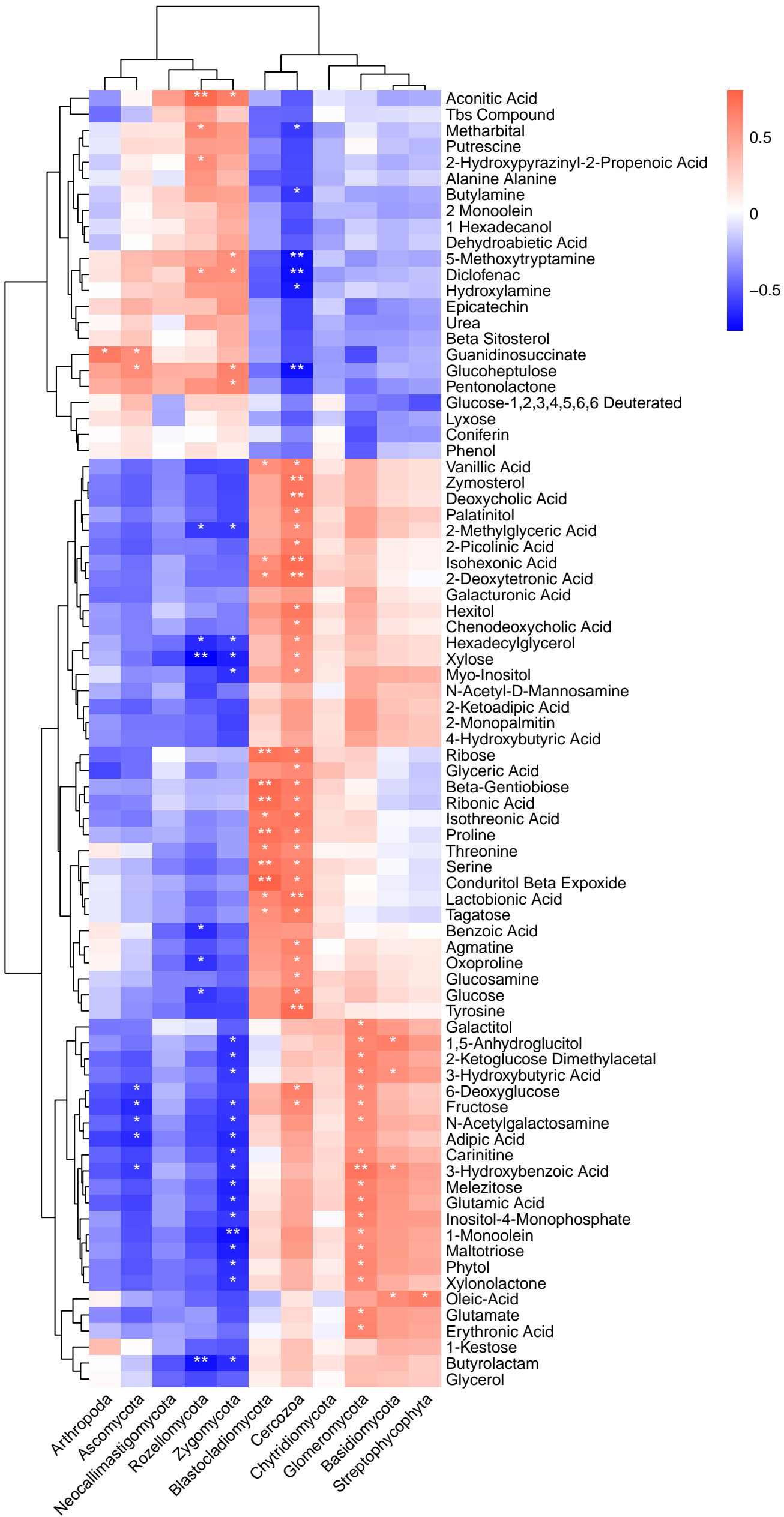

Supplement: Supplementary file 1 [file ijms-23-14707-s001.zip › ijms-2006527-supplementary/Supplementary files/Figure S5 its_phylum_cor_heatmap.pdf]

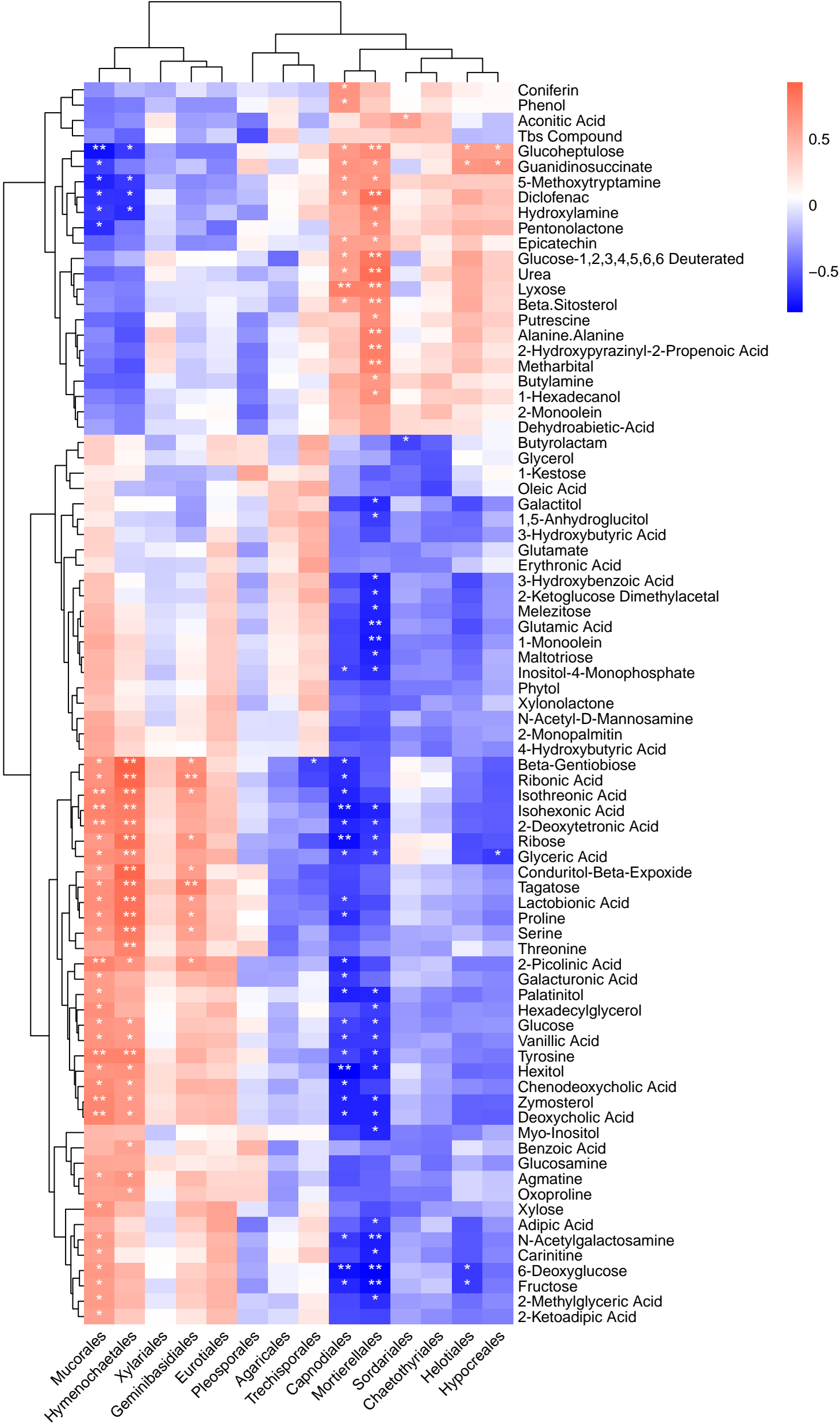

Supplement: Supplementary file 1 [file ijms-23-14707-s001.zip › ijms-2006527-supplementary/Supplementary files/Figure S6 its_order_cor_heatmap.pdf]
